# The associations between epilepsy, metabolism, and their clinical implications

**DOI:** 10.3389/fendo.2026.1694550

**Published:** 2026-01-27

**Authors:** Juan Li, Yiqing Mao, Haiqing Zhang, Xin Xu

**Affiliations:** 1 Department of Neurology, Chongqing Key Laboratory of Neurology, The First Affiliated Hospital of Chongqing Medical University, Chongqing, China; 2 Department of Neurology, Affiliated Hospital of Zunyi Medical University, Zunyi, Guizhou, China

**Keywords:** epilepsy, metabolites, metabolic disease, children, metabolomics

## Abstract

Epilepsy can cause metabolic disorders, and metabolic abnormalities can also trigger epilepsy, forming a bidirectional pathological cycle. Over the past century, from the earliest use of ketogenic diets to treat epilepsy, it has been confirmed that metabolic intervention can control seizures. Subsequent studies have gradually revealed that metabolic disorders such as glucose abnormality and vitamin B6 deficiency can directly induce epilepsy, while epileptic seizures themselves can cause lactic acidosis, electrolyte imbalance and other internal environment disorders. With the breakthroughs in metabolomics technology, the research on epilepsy and metabolism has entered a systematic stage, and their relationship has attracted increasing attention. However, current reviews mostly focus on the isolated analysis of a single metabolic element (such as iron, vitamin D), lacking a systematic integration of multiple metabolic elements. This review for the first time integrates the changes of seven major metabolic elements (glucose, lipids, vitamins, minerals, water, adenosine triphosphate, uric acid) in the onset, progression and treatment of epilepsy; summarizes the clinical associations between metabolic diseases (diabetes mellitus, alcoholism, uremia) and epilepsy; reveals the specific metabolic changes in childhood epilepsy; and emphasizes the importance of epilepsy metabolomics data. It provides a reference for basic research and a metabolic monitoring framework for clinicians.

## 1 Introduction

Epilepsy is a common chronic neurological disorder, with approximately 50 million people living with epilepsy worldwide and 5 million new cases each year. Compared with the general population, people with epilepsy have a 3-fold greater risk of premature death (1).

Epilepsy is a brain network disorder centered around the cerebrum (2). The brain is an organ that is highly dependent on energy, but it does not have sufficient energy reserves of its own; therefore, the brain depends on the metabolism of various exogenous energy substrates to maintain normal function. The relationship between brain metabolism and seizures is complex and bidirectional, as both energy to sustain prolonged seizures and energy to prevent seizures, recover from seizures and repair existing brain damage are required (3).

**Figure 1:**
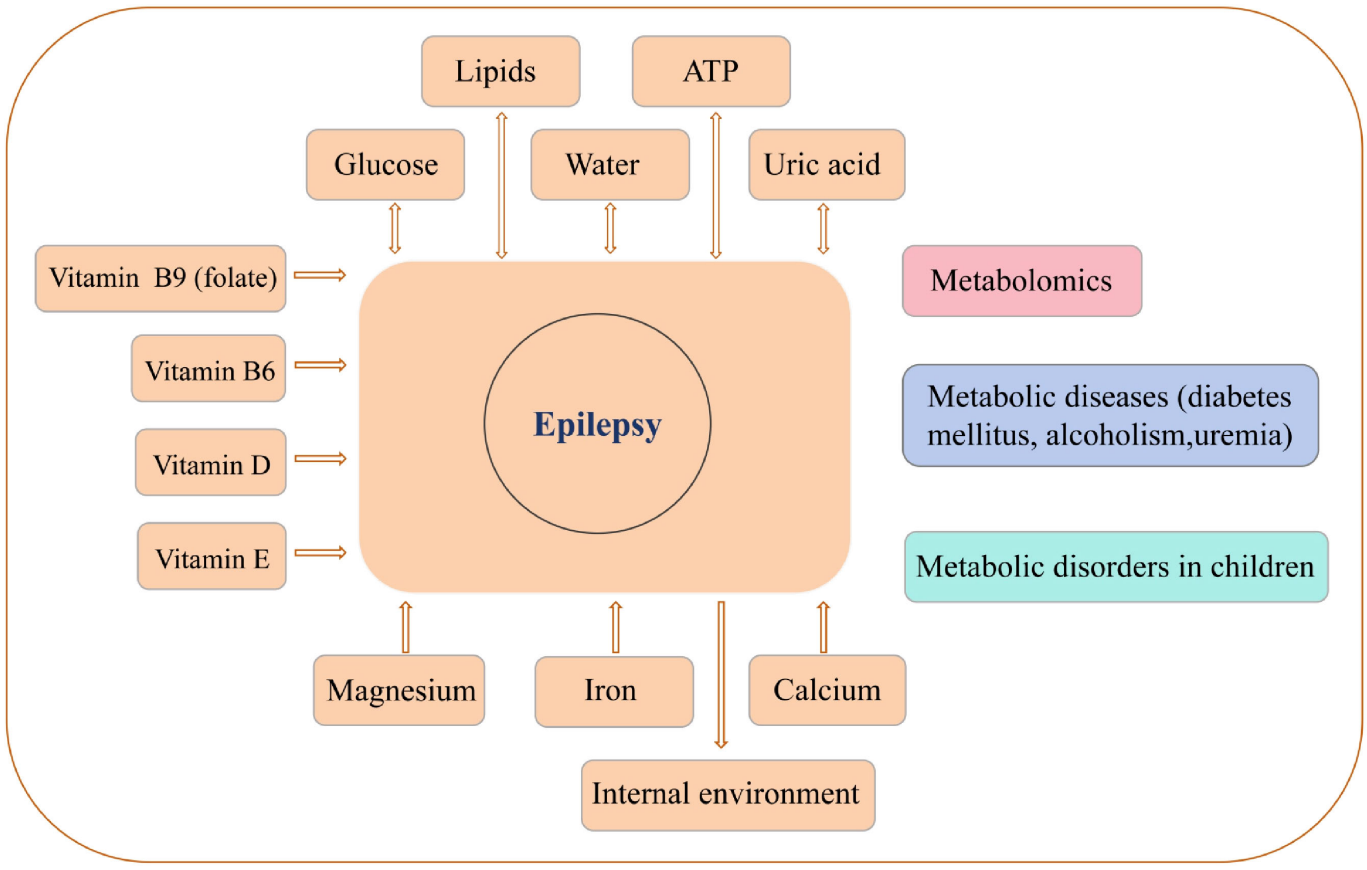
The associations between epilepsy and metabolism. Epilepsy and metabolism interact bidirectionally. Metabolic substances including molecular basis for epileptogenic neurodegeneration. Regarding minerals, iron and intracellular calcium mediate epileptogenesis. Interestingly, magnesium deficiency correlates with seizure severity. Furthermore, epileptic seizures can induce acid-base imbalance in the body (4–74).

Exploring the relationships among the metabolism of various substances, seizures and antiseizure medications (ASMs) and providing new ideas for antiseizure therapy are highly important. Here, the metabolic factors that are grouped and depicted in **Figure 1**. Meanwhile, to provide historical context, **Table 1** presents a timeline of seminal discoveries for key metabolic factors in epilepsy.

**Table 1:** Timeline of key historical insights into metabolic factors in epilepsy (4–14).

Metabolic factor	Year	Representative author	First reported insight
Glucose metabolism	1946	N.G. Hulbert et al.	Hypoglycemia was proposed as a trigger for late-onset epileptic seizures, even in individuals without a prior history of epilepsy.
Lipids metabolism	1921	Wilder	The ketogenic diet was first introduced as a therapeutic approach for the treatment of epilepsy
Folate	1958	I. Chanarin et al.	Antiseizure medication–associated impairment of folate metabolism was first identified.
Vitamin B6	1962	R. Garty et al.	Pyridoxine-dependent infantile seizures were described, establishing vitamin B6 as an essential therapeutic factor.
Vitamin D	1970	C.E. Dent et al.	Long-term use of antiseizure medications was linked to vitamin D inactivation and bone disease.
Vitamin E	1979	O. Ogunmekan	Reduced serum vitamin E levels were reported in children receiving antiseizure medications.
Water metabolism	1931	H. Hopkins-Detrick	Alterations in water and calcium metabolism were implicated in the pathophysiology of epilepsy.
Magnesium	1947	A.D. Lazovik et al.	Magnesium deficiency was shown to induce seizures and increase seizure severity.
Iron metabolism	1988	Z.H. Zhang et al.	Iron-induced lipid peroxidation was proposed as a mechanism contributing to epileptic activity.
Energy metabolism	1970	Sanders et al.	Epileptic seizures were first linked to alterations in cerebral ATP levels.
Uric acid	1975	D.J. Warren et al.	Elevated serum uric acid levels were observed following status epilepticus or recurrent seizures.

## 2 Methods

We conducted comprehensive electronic searches in the PubMed database to identify relevant studies for this review. The search was performed in two distinct time periods: 1931–1989 and 2014–January 2026, given the historical depth of the first period and the focus on recent advancements in the second. These intervals were selected to balance foundational knowledge with cutting-edge research. The primary focus of the search was on epilepsy or seizures related to metabolic, nutritional, and physiological factors. The complete search syntax followed this structure:

(“epilepsy” OR “seizure”) AND (“metabolism” OR “lipid” OR “triglyceride” OR “vitamin” OR “water” OR “mineral” OR “internal environment” OR “energy” OR “uric acid” OR “diabetes” OR “alcoholism” OR “uremia” OR “children” OR “epilepsy metabolomics”).

## 3 Epilepsy and metabolites

### 3.1 Epilepsy and glucose metabolism

In 1946, N.G. Hulbert et al. first proposed that late-onset epilepsy may be related to hypoglycemia, and patients without a history of epilepsy may have seizures that are induced by hypoglycemia (4). In 1977, K.A. Flügel proposed that hyperosmolar nonketoic acid toxicity hyperglycemia can induce focal epileptic activity (15). In 1986, M. Ingvar proposed that persistent neuronal hyperexcitation with an increased glucose metabolism rate is a prerequisite for the occurrence of neuronal injury (16).

In animal models, there is a causal relationship between altered brain glucose metabolism and seizures. In 2017, Evgeniya Samokhina et al. injected the nonmetabolizable glucose analogue 2-deoxy-D-glucose in the lateral ventricle to establish chronic low-energy metabolism models and reported that chronic suppression of brain energy metabolism, especially a reduction in glucose use, can induce epileptic seizures (17). In 2018, Anton Malkov et al. reported that seizures are followed by long-term glucose hypometabolism (18). In 2020, Tanya McDonald et al. reported that reducing glycogenolysis and increasing glucose utilization and metabolism through the tricarboxylic acid (TCA) cycle can play an antiseizure role in the epileptic brain region of a pilocarpine-induced epilepsy mouse model (19). A 2024 study by Marion Bankstahl et al. revealed that increased glucose metabolism was associated with decreased seizure frequency (SF) during the chronic phase of epilepsy (20).

**Figure 2:**
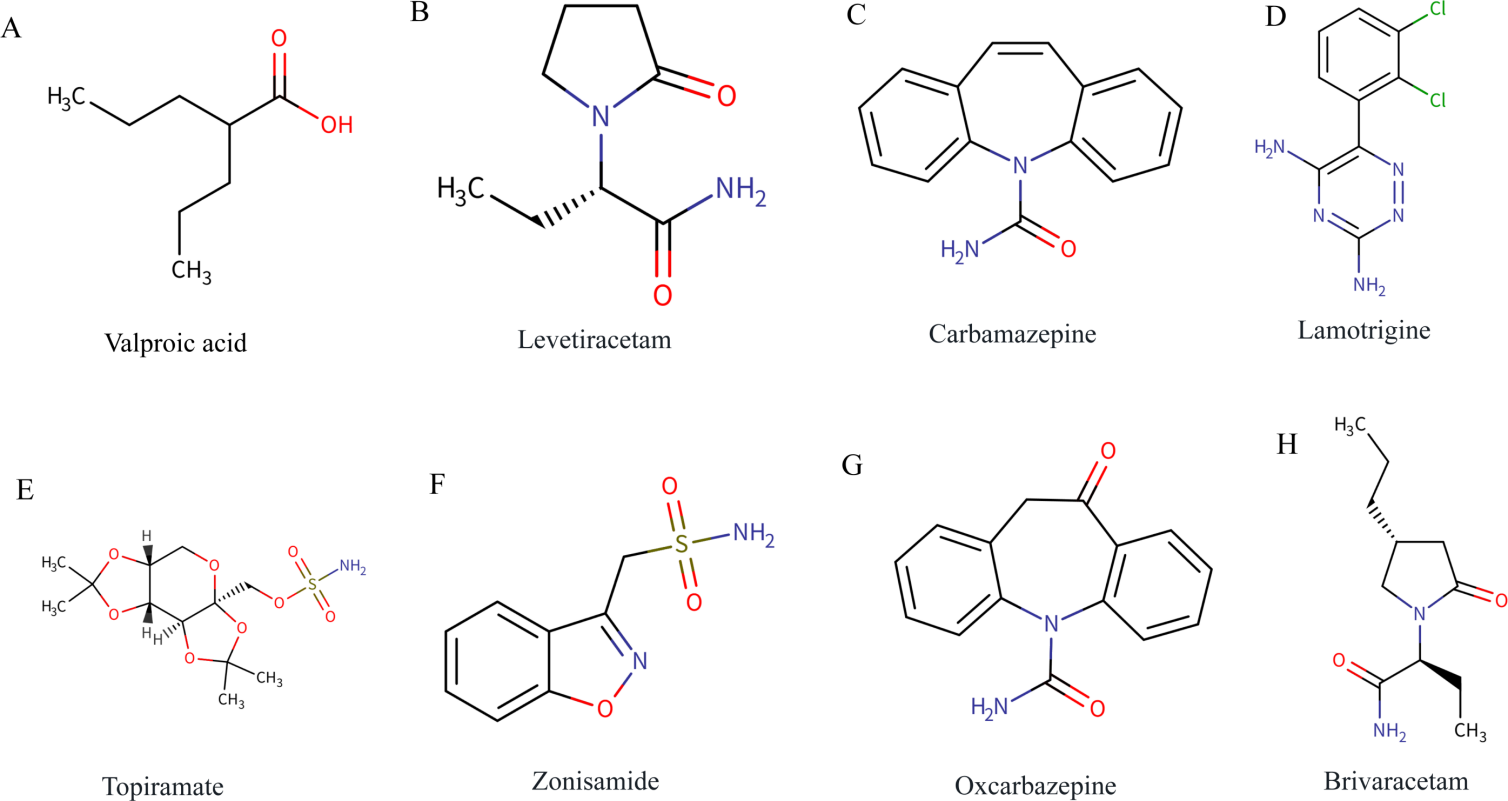
Chemical structures of the eight ASMs involved in this study. **(A)** Valproic acid, **(B)** Levetiracetam, **(C)** Carbamazepine, **(D)** Lamotrigine, **(E)** Topiramate, **(F)** Zonisamide, **(G)** Oxcarbazepine, **(H)** Brivaracetam. The structures were retrieved from the DrugBank Online database (https://go.drugbank.com) (151) and compiled into a single composite figure. Individual drug entries (accession numbers: DB00313, DB01202, DB00564, DB00555, DB00273, DB00909, DB00776, DB05541) were accessed on December 29, 2025.

Antiseizure therapy causes changes in glucose metabolism. For example, during chronic (> 8 months) cervical vagus nerve stimulation for the treatment of drug-resistant epilepsy (DRE), fasting blood glucose levels are significantly higher than baseline levels (21). Valproate (VPA, the chemical structure is shown in **Figure 2A**) can have acute hypoglycemic effects when used in the treatment of epilepsy (22). Patients with epilepsy treated with VPA for a long time (> 6 months) develop insulin resistance and metabolic disorders; however, patients receiving long-term treatment (> 6 months) with levetiracetam (LEV, the chemical structure is shown in **Figure 2B**) do not (23).

### 3.2 Epilepsy and lipids metabolism

In 1921, Wilder first recommended a ketogenic diet to children with epilepsy (5). In 1989, R.H. Schwartz et al. evaluated the short-term clinical effects of three different ketogenic diets on the symptoms of epilepsy, with the type of diet recommended being independent of seizure type. The researchers found that after 3 months of following a ketogenic diet, patients had a reduced SF, and the efficacy in children was better than that in adults (24). In a 2020 prospective study of 8 adults with DRE treated with a modified Atkins ketogenic diet, Gabriela de Souza Neves et al. reported that the number of episodes of impaired consciousness decreased by 55.5% over 12 weeks, but total cholesterol, low-density lipoprotein, and non-high-density lipoprotein levels gradually increased with long-term consumption of this ketogenic diet (25).

Patients with epilepsy exhibit increased levels of glycolipid, inflammatory, oxidative, and apoptotic factors, especially patients with generalized seizures (26). Malondialdehyde levels are also markedly elevated in patients with epilepsy (27).

ASMs affects lipid metabolism. Treatment with VPA negatively affects the expression of lipid mediators, progressively disrupting the function of lipid molecules (28). In a study of elderly people with epilepsy, carbamazepine (CBZ, the chemical structure is shown in **Figure 2C**) significantly interfered with the ability of statins to lower total cholesterol, and lamotrigine (the chemical structure is shown in **Figure 2D**) and LEV had no significant effect on the efficacy of statins (29). However, lamotrigine reduces plasma lipid peroxide concentrations (30).

### 3.3 Epilepsy and vitamins metabolism

#### 3.3.1 Epilepsy and folate

In 1958, I. Chanarin et al. first proposed that primidone affects folate metabolism (6). In 1970, C.D. Allen proposed that vitamin B12 and folate play important roles in maintaining brain activity (31). In 1973, E.H. Reynolds proposed that folic acid and its derivatives have significant antiseizure properties (32).

An increasing number of scholars have recognized the importance of folic acid supplementation in pregnant women with epilepsy. High ASMs concentrations are correlated with high concentrations of unmetabolized folic acid and inactive folate metabolites in epileptic pregnant women (33). In pregnant women with epilepsy who take ASMs, perinatal folic acid use is associated with better cognitive development in their children, as reflected by their Full-Scale Intelligence Quotient at 3 and 6 years of age (34). In a retrospective observational study of 8695 pregnant women treated with ASMs (monotherapy or combination), approximately half of pregnant women in Denmark, Norway and Sweden received high-dose folic acid (≥ 1 mg/day) supplements, but many did not start taking the folic acid supplements until after pregnancy (35). However, an increased risk of cancer has also been reported in children born to mothers with epilepsy who took high doses of folic acid (>5 mg/day) prenatally (36).

#### 3.3.2 Epilepsy and vitamin B6

In 1946, J.T. FOX et al. first proposed that vitamin B6 (pyridoxine) has no obvious effect when used in the treatment of epilepsy (37). In 1962, R. Garty et al. reported cases of pyridoxine-dependent convulsions in infants, for which ASMs other than pyridoxine were ineffective (7).

Vitamin B6 has a positive regulatory effect on ASMs. For male veterans who use LEV for antiseizure therapy, vitamin B6 supplementation may help reduce LEV-related irritability (38). Vitamin B6 supplementation also significantly reduces the likelihood of drug discontinuation due to behavioral abnormalities caused by LEV in children with epilepsy (39). High ASMs concentrations in pregnant women with epilepsy are associated with the presence of metabolically active pyridoxine (33).

#### 3.3.3 Epilepsy and vitamin D

In 1970, C.E. Dent et al. reported for the first time that osteomalacia occurred in 4 patients who used phenytoin, primidone, and phenobarbitone for long-term antiseizure therapy, possibly because drug-mediated enzyme induction led to a significant increase in the degree of vitamin D inactivation (8). In 1973, C. Christiansen et al. reported a significant increase in the bone mass of epilepsy patients receiving long-term phenytoin sodium combined with vitamin D supplements (40). In 1975, R. Bouillon found that patients treated with ASMs (barbiturates and diphenylhydantoin, with six patients receiving additional ASMs) had vitamin D deficiency, which led to hypocalcemia and secondary hyperparathyroidism (41).

The relationship between vitamin D supplementation and SF in patients with DRE is controversial. The prevalence of vitamin D deficiency is high in epilepsy patients, and ASMs can reduce vitamin D levels in epilepsy patients (42). In 2024, Francine Chassoux et al. studied DRE and vitamin D deficiency in patients aged 15 years or older. Long-term vitamin D3 supplementation to achieve optimal 25-hydroxyvitamin D (25(OH)D) levels reduced the SF and the frequency of bilateral tonic–clonic seizures (43). On the basis of these findings, monitoring of vitamin D levels has been recommended as part of the routine management of patients with epilepsy (42). However, two independent studies (2018, 2025) failed to observe a reduction in SF with vitamin D supplementation in adults with DRE (44, 45). Despite the differences in the findings of these studies (**Table 2**), the results do suggest that long-term (≥6 months) and adequate-dose vitamin D supplementation may reduce SF in patients with DRE, but this requires confirmation in larger, prospective trials.

**Table 2:** Summary of vitamin D supplementation protocols and effects on seizure frequency.

References	Intervention protocol & timeline	Main finding (seizure frequency)
(43)	Dosage:100,000 IU per month Duration:6 months Assessment:① At the end of treatment (6 months); ② Post-treatment follow-up at 6 months (12 months)	Median reduction of 30% and 33%, respectively.
(43)	Dosage: 100,000 IU × 5 doses (within 3 months) Duration: 3 months Assessment: At the end of treatment (3 months)	No significant reduction.
(44)	Dosage (adjusted by baseline status): • Deficiency: 100,000 IU/week • Insufficiency: 100,000 IU twice per month Duration: 3 months Assessment: Post-treatment follow-up at 3 months (6 months)	No significant reduction.
(45)	Dosage:​ Loading phase: 60,000 IU/week for 3 months Maintenance phase: 60,000 IU/month for 3 months Duration: 6 months Assessment: Monthly from baseline until the end of treatment	No significant reduction.

#### 3.3.4 Epilepsy and vitamin E

In 1979, O. Ogunmekan first proposed that the plasma vitamin E level of children with epilepsy who took ASMs (either singly or in combination but with no other drugs, particularly iron) between 2 and 12 years of age was lower than that of healthy children (9). Vitamin E serves as the molecular basis of epileptogenic neurodegeneration and is involved in excitotoxicity, neuroinflammation and lipid peroxidation (46). Clinical data suggest that some antioxidants (melatonin, selenium, and vitamin E) may be recommended as adjunctive therapies for patients with DRE (47).

In an epilepsy model, vitamin E was involved in seizures and improved bone tissue metabolism. By inhibiting the expression of lipoxygenase 15 (48), vitamin E ameliorated the dysfunctional metabolism of calcium, magnesium and zinc in the deteriorated bone tissue of epileptic rats (49); although long-term monotherapy with vitamin E reduced oxidative stress, SF was not reduced (50).

### 3.4 Epilepsy and metabolism of other substances

#### 3.4.1 Epilepsy and water

In 1931, H. Hopkins-Detrick first explained the influence of calcium and water metabolism on the occurrence and development of epilepsy and mentioned that fluid retention in brain tissue is an important symptom of epilepsy and that dehydration has unique value in its treatment (10). In 1955, N.A. Bercel proposed that the addition of diuretics could reduce the toxicity of the ASMs phenobarbital and Tridione (51). In 1988, L. Vivalda et al. monitored blood sodium, blood potassium, plasma and urine osmotic pressure and free water clearance in 27 patients taking CBZ and reported that there were no complications related to water retention during oral CBZ treatment (52).

Water itself as a medium can induce epilepsy. In 2015, researchers reported that bathing epilepsy, caused by showering or bathing (regardless of water temperature), is a type of reflex epilepsy caused mainly by unique genetic mutations; other triggers include cutting nails, cutting hair, or watching others bathe (53). Hot water epilepsy, induced by contact with hot water, has also been reported by several scholars (54, 55).

#### 3.4.2 Epilepsy and minerals

Seizure severity and SF are associated with serum magnesium levels, with lower levels leading to more severe seizures and higher SF. In 1947, A.D. Lazovik et al. fed rats a magnesium-deficient diet, and 17 out of 18 magnesium-deficient animals were prone to seizures; this was the first suggestion that the severity of seizures increased with a continuous decrease in magnesium levels (11). A Bangladeshi study of 55 patients with idiopathic generalized epilepsy revealed that patient serum magnesium levels were lower than those of healthy controls and that the mean serum magnesium levels were significantly lower in patients with more monthly seizures (>3) (56). A prospective study of dietary magnesium intake and the incidence of epilepsy in 2442 middle-aged and older men in Finland revealed that higher dietary magnesium intake was associated with a lower incidence of epilepsy (57).

Iron-induced lipid peroxidation is an important mechanism of neuron death in patients with epilepsy. In 1988, Z.H. Zhang et al. proposed that iron-induced lipid peroxidation impaired γ-aminobutyric acid (GABA) release and uptake, leading to GABA inhibition as a possible neurochemical pathogenesis of iron-induced epilepsy (12). In 2021, T.S Zimmer et al. proposed that epilepsy-mediated chronic neuronal iron uptake may play a role in neuronal dysfunction or loss in temporal lobe epilepsy (TLE) and hippocampal sclerosis (58). In 2024, Yang Su et al. reported that ferroptosis is an important neuron death mechanism in TLE; ferroptosis is caused mainly by lipid accumulation and oxidative stress (59).

Maintaining intracellular calcium homeostasis is a new research direction for the prevention and treatment of epilepsy. Intracellular calcium homeostasis and its dysregulation are the basis of seizures, and the prevention of calcium dysregulation due to reperfusion injury may be an effective approach to treat acute symptomatic seizures and reduce the risk of epilepsy after acquired stroke (60). Validation studies of local environmental changes in Ca^2+^ and pH within astrocytes in the hippocampus of animals with epilepsy revealed that all cellular enzyme responses are affected by Ca^2+^ and pH and that controlling astrocyte pH and/or Ca^2+^ may be a new target for epilepsy therapy or the prevention of epilepsy-related adverse events (61).

Antiseizure therapy is associated with electrolyte levels. LEV significantly reduces blood calcium levels and affects bone metabolism in patients with epilepsy (62). Iron supplementation during CBZ administration may result in reduced CBZ absorption (63). Decreased serum levels of trace elements (zinc and copper) may be associated with epilepsy, and trace mineral supplementation may reduce the risk of epilepsy (27).

#### 3.4.3 Epilepsy and internal environment

Acidosis caused by epileptic seizures has no significant effect on prognosis, but metabolic alkalosis is associated with poor prognosis. In 1975, B.R. Brooks et al. reported that lactic acid levels in arterial blood and cerebrospinal fluid (CSF) increased after epileptic seizures, but the levels of both returned to normal after 4 days (64). Analysis of arterial blood gas within 24 h after status epilepticus revealed acid–base imbalance in half of the patients, and metabolic acidosis and respiratory acidosis were the most common but had no significant effect on prognosis. However, 6% of metabolic alkalosis cases were associated with increased in-hospital mortality, and 9% of metabolic alkalosis cases were associated with adverse functional outcomes (65). Topiramate (the chemical structure is shown in **Figure 2E**) and zonisamide (the chemical structure is shown in **Figure 2F**) cause metabolic acidosis before, during, and after surgery (craniotomy in patients with epilepsy) (66).

#### 3.4.4 Epilepsy and adenosine triphosphate

Sanders et al. (1970) proposed that epileptic seizures are related to the ATP concentration in the brain (13). In 1981, M.I. Ingvar et al. reported that the hippocampus metabolic rate increased as epilepsy progressed (67).

The brain energy efficiency of patients with TLE was found to be significantly lower than that of healthy volunteers, especially on the focal side (68). The metabolic characteristics of patients with mesial TLE and patients with neocortical TLE were analyzed, and the two epilepsy types could be distinguished on the basis of differences in metabolism in each brain region (69). In the brain sections of epileptic rats, acute epileptic activity promoted ATP production, whereas chronic epileptic activity decreased ATP production capacity by 25% to 40% (70).

#### 3.4.5 Epilepsy and uric acid

In 1975, D.J. Warren et al. first reported high serum uric acid levels in patients with epileptic status or recurrent seizures (14). In the same year, O.D. Saugstad proposed that elevated blood uric acid levels after seizures were due to tissue hypoxia during seizures, accelerating purine catabolism, and recommended routine measurement of hypoxanthine to evaluate tissue hypoxia after seizures (71). In 1989, R.M. Schwartz et al. proposed that a ketogenic diet can increase plasma uric acid levels (72).

Low blood uric acid levels are associated with the risk of epilepsy and depressive symptoms. A cross-sectional study using 2013–2018 National Health and Nutrition Examination Survey data revealed that low serum uric acid levels in adults were significantly associated with the risk of epilepsy and may be an independent risk factor for epilepsy (73). In epilepsy patients ≥18 years of age, lower serum uric acid levels were associated with depressive symptoms (74). In a mouse model of epilepsy, disruption of urate oxidase activity reduced susceptibility to pentatetrazole- and pilocarpine-induced epilepsy, possibly through a chronic increase in uric acid levels leading to decreased brain excitability (75).

## 4 Epilepsy and metabolic diseases

### 4.1 Epilepsy and diabetes mellitus

#### 4.1.1 Epilepsy and diabetes mellitus correlation

In a retrospective study of 229 adults with type 1 diabetes mellitus (T1DM) in Spain, the prevalence of epilepsy was 2/229 (0.8%) (76). In Taiwan and the United Kingdom, T1DM population cohort studies revealed that the risk of epilepsy in the T1DM population was approximately 3 times greater than that in the control population (77, 78). In a longitudinal study of patients with T1DM in Taiwan, the onset of epilepsy in patients with T1DM was related to age. The incidence of epilepsy in adulthood (> 18 years old) is 2.26 times higher than that in childhood (≤18 years old). An age range of disease onset of 30–40 years, male sex, more than 1 diabetic ketoacidosis seizure, and idiopathic seizures were independent risk factors for epilepsy after the onset of T1DM (79).In a longitudinal study of 6,162 children with previously diagnosed TIDM in Finland, the incidence of new-onset epilepsy was greater in children with TIDM than in the control group (the risk per 100,000 person-years was 140 and 82, respectively), but the incidence of TIDM gradually increased, and the risk of epilepsy gradually decreased over time (80).

In a 10-year follow-up study of 751,792 type 2 diabetes mellitus (T2DM) patients in Taiwan, the incidence of epilepsy was higher in T2DM patients than in controls (35.0 and 21.9 per 10,000 person-years, respectively), and T2DM increased the risk of epilepsy by approximately 50% (81). A retrospective study of a Taiwanese population revealed that patients with epilepsy had a greater risk of T2DM than controls did, with an adjusted hazard ratio (aHR) of 1.27. Patients with epilepsy who were not treated with ASMs had a significantly greater risk of T2DM than nonepileptic controls did (aHR, 1.70), and patients who were treated with ASMs had a significantly lower risk of T2DM than those who were not treated (all aHR ≤0.60) did (82). A large-scale whole-genome meta-analysis of serious autoimmune T2DM (research object = 452, control = 2,744) and focal epilepsy (research object = 929, control = 212,532) in Europeans revealed that genetic susceptibility to severe autoimmune T2DM was associated with an increased risk of focal epilepsy (83). Long-term use of enzyme-inhibiting ASMs was associated with an increased risk of new-onset T2DM (84).

#### 4.1.2 Clinical characteristics of patients with epilepsy complicated with diabetes mellitus

Among 2016 patients with DRE, 20 patients with T1DM had focal epilepsy, with most seizures originating in the temporal lobe; in 80% of these patients, T1DM predated the onset of epilepsy by a median time of 1.5 years (85). The genetic characteristics of epilepsy combined with diabetes mellitus have also been reported by many scholars. tRNA methyltransferase homologous A gene mutation causes an autosomal-recessive genetic disorder commonly characterized by microcephaly, diabetes mellitus, and epilepsy. This disorder is characterized by normal head magnetic resonance imaging (MRI) findings but an ictal electroencephalogram (EEG) showing a rhythmic 5 Hz theta frequency, beginning in a large area in the left hemisphere, and an interictal EEG showing both parasagittal sinus area and systemic spike wave discharges in stage two sleep (86). ATP-sensitive potassium gain-of-function mutations and mutations in the mitochondrial asparaginyl-tRNA synthetase 2 gene can manifest as developmental delay, epilepsy, and neonatal diabetes syndrome (87, 88).

#### 4.1.3 Diabetes mellitus link to epilepsy

Diabetes mellitus associated with epilepsy involves multiple molecular mechanisms. Among these, dysglycemia is an important predisposing factor: studies have shown that chronic inhibition of cerebral energy metabolism, especially reduced glucose utilization, can induce epileptic seizures (17), while decreasing glycogenolysis and increasing glucose utilization and metabolism via the TCA cycle exerts an antiseizure effect (19). Notably, neonatal hypoglycemia significantly increases the risk of epilepsy in later childhood (89). Mitochondrial dysfunction and adiponectin deficiency may be one of the mechanisms contributing to the comorbidity of epilepsy and T2DM (90). The inflammatory mechanism plays a role in the development of epilepsy associated with T2DM, as inflammatory biomarkers such as interleukins (IL-1β, IL-6, and IL-8), tumor necrosis factor-α, high-mobility group box 1, and toll-like receptors are elevated in both epileptic seizures and T2DM (91). The autoimmune mechanism is a key linking pathway specifically in the association between T1DM and epilepsy: glutamic acid decarboxylase antibodies play a central role in this connection, and translocator protein, a differentially expressed gene upregulated in both, may also be involved in this regulatory process (92). Additionally, genetic predisposition to severe autoimmune T2DM is associated with an increased risk of focal epilepsy (83). Genetic factors are equally noteworthy: recessive TMEM167A variants can directly cause neonatal diabetes mellitus, microcephaly, and epilepsy syndrome (93), while epilepsy and T1DM share four potential pathogenic factors: genetic predisposition, factors involved in autoimmune responses, dysglycemia, and ischaemic processes caused by cerebrovascular damage (94). Regarding Diabetes mellitus management, the effect of metformin, a classic anti-diabetic drug, on epilepsy is controversial: on the one hand, studies have confirmed that it, as an AMPK agonist, activates thalamic AMP-activated protein kinase, thereby enhancing metabotropic GABAB receptor function and ultimately promoting absence epileptic seizures (95); on the other hand, other studies have suggested that it alters gut microbiota to favor agmatine production, exerting a neuroprotective effect against epilepsy (96). By contrast, the use of sodium-glucose cotransporter 2 inhibitors is associated with a lower incidence of epilepsy (97). Additionally, ketogenic diets have also gained increasing attention in the treatment of T2DM (98).

### 4.2 Epilepsy and alcoholism

#### 4.2.1 New-onset epilepsy and alcoholism

Acute symptomatic seizures were attributed to acute alcoholism in 2.9% (3/102) of the adults who presented for the first time in the emergency department in five Latin American countries (99). In a study of all new-onset epilepsy patients in North Macedonia aged 20–49 years from 2015 to 2018, 5% of the cases were associated with alcohol abuse (100). Metabolic disorders are responsible for some new seizures in people older than 50 years, 84% of which are caused by chronic alcoholism (101).

#### 4.2.2 Epilepsy and alcohol withdrawal

Alcohol withdrawal was found to be the most common cause of acute symptomatic seizures (74.1%) (102). Eleven percent of patients with alcohol withdrawal seizures experienced relapse, and 35.7% had abnormal brain computerized tomography findings. The death rate was 2.9% per year, which was 13 times higher than that of the general population. In 88.8% of patients, EEGs were normal or showed either diffuse high-frequency patterns or altered alertness, while in 5.1% of patients, EEGs showed interseizure epileptoid discharges and seizures (103).

#### 4.2.3 Epilepsy and alcohol dependence

A study of Polish prisoners revealed that alcohol-dependent prisoners were four times more likely to develop epilepsy than were those without this condition and that alcohol dependence was strongly associated with epilepsy, independent of other variables (104). An analysis of Ireland’s National Drug-Related Deaths Index from 2004 to 2013 revealed that more than two-thirds (31%~70%) of patients whose cause of death was epilepsy were alcohol dependent and had no ASMs in their bodies at the time of death (105).

#### 4.2.4 Alcoholic encephalopathy with epilepsy

In a retrospective analysis of 34 patients with subacute encephalopathy with alcoholic epileptic syndrome, abstaining from alcohol was the cause in 35.2% of patients, excessive alcohol consumption was the cause in 11.7% of patients, 41.1% of patients had a history of seizures in the context of alcohol withdrawal syndrome, 85.2% of patients had a partial periodic discharge observed on an EEG, and 64.7% of patients had a high signal area and limited diffusion in neuroimaging (106). Subacute encephalopathy with seizures in alcoholics syndrome may recur after chronic antiseizure treatment is discontinued (107).

### 4.3 Epilepsy and uremia

#### 4.3.1 Association of uremia with seizures

Patients with end-stage renal disease who underwent dialysis had a greater prevalence of epilepsy (8.8%), and the risk of death in patients diagnosed with epilepsy was 1.11 times greater than that in patients without epilepsy (108). Uremic encephalopathy accounted for 3.9% (4/102) of acute symptomatic seizures in adults presenting for the first time in emergency departments in five Latin American countries (99).

#### 4.3.2 Clinical characteristics of epilepsy caused by uremia

In a study of maintenance hemodialysis patients, new-onset epilepsy was often severe and progressed rapidly, and the mortality rate was high (45.71%, 16/35). Female dialysis patients with infection, taking antibiotics and with hypoalbuminemia were more likely to have epilepsy (109). In 2017, Min-Surk Kye et al. reported a case of aphasic status epilepticus related to uremia, characterized by repeated episodes of aphasia but no other cognitive impairments. MRI revealed no significant lesions, and analysis of an EEG revealed ictal discharges in the left frontotemporal lobe. The aphasic status epilepticus was cured after intravenous VPA and uremia correction (110). In 2023, Chongyang Han et al. reported 2 cases of epilepsy in dialysis patients, one of which was accompanied by reversible posterior leukoencephalopathy syndrome (RPLS), and the RPLS subsided rapidly after treatment. Epilepsy symptoms were also controlled (111).

## 5 Association of epilepsy with metabolic disorders in children

### 5.1 Congenital metabolic disorders and epilepsy in children

In 7% of children, epilepsy is caused by a congenital metabolic disorder (112). In 1954, A.D. HUNT Jr et al. first reported a case of intractable convulsion in an infant dependent on pyridoxine (113). In 1964, B. Hagberg et al. reported that three children with epilepsy with tryptophan metabolism disorders were treated with vitamin B6, and their symptoms were significantly alleviated (114). In 2017, Suvasini Sharma et al. listed common congenital metabolic disorders causing epilepsy, such as mitochondrial diseases, glucose transporter-1 deficiency, and congenital and early infantile neuronal ceroid lipofuscinosis, on the basis of case reports (115).

### 5.2 Metabolic characteristics of children with epilepsy

Brain glucose hypometabolism is negatively correlated with SF, and poor glucose metabolism affects child development. Measurement of glucose metabolism in the cerebral lobes of 41 children with DRE during the interseizure period revealed that 63% of the children had an enlarged interval of low metabolism, and 68% of the children had developmental delay. The expanded range of metabolic dysfunction in children with persistent frequent seizures is associated with developmental delay, and if surgical treatment is not available, medical treatment to control seizures may improve neurocognitive outcomes (116). Compared with patients with drug-sensitive epilepsy, patients with DRE showed significant widespread and progressive poor glucose metabolism in the brain (117). The absolute asymmetry index of abnormal cerebral glucose metabolism in children with epilepsy was markedly positively correlated with the SF and negatively correlated with the time to the last seizure. The absolute asymmetry index value in DRE patients was greater than that in patients with epilepsy remission (118).

Vitamin D supplementation in children with epilepsy may have a role in controlling epilepsy. Some scholars have proposed that children with epilepsy who take ASMs need to take vitamin D supplements daily, especially those who receive monotherapy; 1000 IU/d may be beneficial to children and may have the effect of controlling epilepsy (119). In a retrospective study of serum 25(OH)D levels in 648 children with epilepsy in China, the serum 25(OH)D level in children with epilepsy was reduced both before and during ASMs treatment, and the SF was significantly reduced in the group treated with ASMs and receiving vitamin D supplements (120). However, excessive maternal vitamin D levels during pregnancy, resulting in high 25-hydroxyvitamin D3 (25(OH)D_3_) in newborns, is associated with an increased risk of childhood epilepsy (epilepsy first diagnosed at 1–4 years of age) (121).

### 5.3 Dietary therapy for children with epilepsy

A ketogenic diet was first recommended for the treatment of epilepsy by Wilder in 1921, and ketogenic diets were widely recommended for children with epilepsy between 1921 and 1930 (5). In 1971, P.R. Huttenlocher et al. proposed a diet high in medium-chain triglycerides as a treatment regimen for DRE in children; this diet has fewer restrictions on carbohydrates, better taste and easier preparation than the standard ketogenic diet and may have superior antiseizure effects. This diet also appears to be particularly effective in children with mild motor and myoclonic epilepsy (122). In 2020, Vishal Sondhi et al. reported that, in 158 children with DRE, a low-glycemic-index diet had significantly fewer adverse effects than did a ketogenic diet or a modified Atkins ketogenic diet (123). In 2023, Antonio Corsello et al. proposed that long-term ketogenic diets in children and adolescents may affect growth and nutritional status, suggesting that prophylactic use of micronutrient supplements should be considered before starting any ketogenic diet (124).

### 5.4 Metabolic effects of ASMs on children

Long-term sodium VPA use had significant negative effects on bone mineral density and vitamin D levels in children with epilepsy (125). Hyponatremia is an adverse effect of oxcarbazepine (the chemical structure is shown in **Figure 2G**) treatment, and daily oral sodium chloride in children with epilepsy treated with monotherapy can safely and effectively reduce the incidence of hyponatremia (126). CBZ monotherapy may lead to significant reductions in serum folic acid and vitamin B12 levels in children with epilepsy (127). There were no significant changes in serum sodium, potassium, or magnesium levels in children with epilepsy treated with LEV monotherapy at 2 and 6 months (128). Treatment with brivaracetam (the chemical structure is shown in **Figure 2H**) had no significant long-term effect on body weight in children with epilepsy (129). In children with self-limiting epilepsy with central temporal spikes, epilepsy itself can affect multiple aspects of bone metabolism; oxcarbazepine, LEV and topiramate do not significantly affect bone metabolism; and lamotrigine can correct some abnormal markers of bone metabolism (130).

## 6 Epilepsy metabolomics

### 6.1 Metabolomic analysis for epilepsy diagnosis

An increasing number of researchers are exploring new biomarkers for the diagnosis of epilepsy via metabolomic analysis. Studies on serum tryptophan, nicotine, linoleic acid, purine and other metabolic pathways have revealed significant differences, particularly in the tryptophan metabolic pathway. Plasma 2S,6S-/2S,6R-oxopropylpiperidine-2-carboxylic acid (2-OPP); 6-oxopiperidine-2-carboxylic acid (6-oxoPIP); 6-hydroxy-2-aminocaproic acid (HACA), an isomer of C9H11NO4; and piperideine-6-carboxylate (P6C), a diastereoisomer derivative, are suitable biomarkers for pyridoxine-dependent epilepsy (131–139). The details are presented in **Table 3**.

**Table 3:** Study of potential diagnostic biomarkers for epilepsy.

References	Experimental group size (n)	Age (mean ± SD), years	Diagnosis	Control group size (n)	Age (mean ± SD), years	Type	Methods	Materials	Potential biomarkers
(131)	9	3-16 (age of operation)	DRE	None	None	None	High-resolution magic angle spinning proton magnetic resonance spectroscopy, cDNA microarrays, and histological analysis	Cortical regions	Reduced lactate and increased creatine, phosphocreatine and choline
(132)	22	18-60	Epilepsy	10	Not described	Healthy people	Liquid chromatography–tandem mass spectrometry based targeted metabolomic analysis	Serum	Tryptophan, kynurenine and 7Z,10Z,13Z,16Z-docosatetraenoic acid
(133)	78	49.9 ± 19.0	SE	107	43.2 ± 17.7	Inpatient without SE	Liquid chromatography coupled with high-resolution mass spectrometry	Plasma, CSF (both groups had 29)	Tryptophan, quinolinic acid
(134)	131	Adult	First-diagnosed epilepsy	527	adult	Healthy people	LC-MS and nontargeted metabolomics detection	Serum	L-alpha-glutamyl-L-hydroxyproline, vulgarone A, MG(0:0/14:1(9Z)/0:0), cis-3-hexenyl phenylacetate
(135)	7	3-28, average 14	PDE	Control brain tissue was acquired from the National Institute of Child Health and Human Development Brain and Tissue Bank for Developmental Disorders at the University of Maryland, Baltimore, Maryland, USA			Untargeted metabolomic analysis and infrared ion spectroscopy	Plasma, CSF and urine	2-OPP and 6-oxoPIP
(136)	9	11 months -31 years old	PDE	22	2months -44 years old	Not described	Ultra high-performance liquid chromatography-high-resolution mass spectrometry and global metabolomic analysis	Plasma	HACA and an isomer of C_9_H_11_NO_4_
(137)	7	Not described	PDE	26	Not described	Not described	High-throughput untargeted LC-MS, targeted molecular identification and infrared ion spectroscopy	Plasma, CSF and urine	Two diastereoisomer derivatives of P6C
(138)	18	7.6 ± 5.04	Epilepsy	11	7 ± 5.14	Healthy children	LC-MS	Plasma	Indole, indoxyl sulfate, p-cresyl sulfate, N1-Methyl-2-pyridone-5-carboxamide (Met2PY) and N1-Methyl-4-pyridone-3-carboxamide (Met4PY)
(139)	15	37.67 ± 15.53	DRE	10	51.60 ± 18.20	Non-epilepsy	LC-MS	Buffy coat	Adenine, L-phenylalanine, 5-methoxyindoleacetate, 2’-deoxyinosine, 3-lsopropylmalic acid
(140)	30	30.8 ± 1.6 and 28.9 ± 1.2	15 with catamenial epilepsy and 15 with TLE	15	29.6 ± 1.8	Healthy people	LC-MS	Fecal and serum	Serum lysophosphatidylinositol
(141)	32	33.7 ± 12.0	Epilepsy	28	33.6 ± 9.9	Healthy people	Untargeted nuclear magnetic resonance spectroscopy	Serum	Citrate, glutamate, proline, 3-methyl-2-oxovalerate, and glucose
(142)	23	8.52 ± 2.57	Refractory epilepsy	10	11.2 ± 3.58	Non-epilepsy	Ultra-high performance LC-MS	CSF (n=16 and 8)	Alpha-ketoisocaproic acid, alpha-ketoisovaleric acid, and acetyl-L-carnitine

SE, status epilepticus; PDE, pyridoxine-dependent epilepsy; 2-OPP, 2S,6S-/2S,6R-oxopropylpiperidine-2-carboxylic acid; 6-oxoPIP, 6-oxopiperidine-2-carboxylic acid; P6C: piperideine-6-carboxylate; LC-MS, liquid chromatography-mass spectrometry.

### 6.2 Association of metabolomics with various metabolites

Metabolomic studies in epilepsy have revealed disturbances in key metabolic pathways involving lipids, glucose, and vitamins. Regarding lipids metabolism, multiple studies have confirmed its close correlation with epilepsy: for example, sphingosine 1-phosphate can protect mitochondrial structure and function to alleviate neuronal hyperexcitability and damage in patients with DRE (143); elevated apolipoprotein E expression in hippocampal microglia drives the progression of TLE by altering glycerophospholipid metabolism (144); the glycerophospholipid pathway is the most enriched in catamenial epilepsy, which may be related to sex hormones (140); octanoic acid in ketogenic diet with medium-chain triglycerides exerts neuroprotective effects on epileptic children by activating G protein-coupled receptor 40 (GPR40) (145). Furthermore, several serum metabolites identified in the metabolomic analysis are enriched in the ferroptosis pathway (132), a form of cell death implicated in epilepsy and linked to lipid peroxidation (59). Acetyl-L-carnitine, a key hub molecule linking lipid metabolism and energy metabolism, also exhibits significant changes in children with DRE (142). In the field of glucose and energy metabolism, metabolomic studies have identified glucose as a differential metabolite with potential biomarker value (141). In TLE, abnormalities in brain network dynamics are associated with specific gene expression, and this association is significantly mediated by changes in cerebral glucose metabolism (146). Abnormal energy metabolism exists in epileptic brain regions (131), while the TCA cycle, a key pathway for energy and glucose metabolism, is significantly abnormal in epilepsy (138). In vitamin-related studies, the metabolic pathways of vitamin A and vitamin C are significantly alterations (138). Notably, vitamin B6-related metabolomic studies focus on the exploration of biomarkers for PDE, and relevant differential metabolites have been detected in biofluids (135–137, 147). In addition, two studies have indicated that purine metabolism is upregulated in epileptic patients (134, 139), with increased serum uric acid levels (134).

### 6.3 Metabolites and ASMs

Women with epilepsy treated with lamotrigine monotherapy showed a trend toward higher circulating 3α-hydroxy-5α/β-reduced androstane levels (148). The plasma levels of 5-methyltetrahydrofolate and tetrahydrofolate decreased with lamotrigine monotherapy during pregnancy, but LEV monotherapy had no effect (149). In patients with epilepsy treated with VPA, the levels of plasma metabolites such as triglycerides, sphingomyelin, phosphorylcholine, ceramides, and phenolic phthiocerols were significantly increased, and the levels of phosphoethanolamines, diacylglycerols, 1α,25-dihydroxy-24-oxo-22-oxavitamin D3, 2-deoxy-20-hydroxy-5alpha-ecdysone 3-acetate, and dolichyl-4 phosphate were significantly decreased (28). In a pentatetrazole-induced mouse model of epilepsy, tryptophan metabolism and phenylalanine metabolism were involved in modulating the effects of berberine in the treatment of epilepsy (150).

## 7 Conclusion and future development directions

Collectively, patients with epilepsy exhibit elevated levels of glycolipids, inflammatory mediators, oxidative stressors, and apoptotic factors, while the pathogenic role of glucose metabolism disorders continues to garner significant attention. Notably, lower serum magnesium levels are robustly associated with increased seizure severity and higher SF, and similarly, hypouricemia in adults demonstrates a significant correlation with epilepsy risk. Mechanistically, iron-induced lipid peroxidation emerges as a key driver of neuronal death, whereas maintaining intracellular calcium homeostasis presents therapeutic potential for seizure control. Clinically, seizure-induced metabolic alkalosis portends a poor prognosis. From these insights, epilepsy metabolomics enables: (1) definition of disease-specific metabolic signatures; (2) discovery of novel diagnostic/therapeutic biomarkers; and (3) quantitative evaluation of ASMs efficacy.

Antiepileptic treatments may induce metabolic alterations including dysglycemia, insulin resistance, and vitamin D deficiency. These agents can concurrently attenuate the lipid-lowering efficacy of statins and compromise bone mineral density, potentially leading to metabolic disorders and secondary hyperparathyroidism. For patients on long-term ASMs, supplementation with folic acid, vitamins B12, B6, and D is recommended, though significant intercountry variations exist in folic acid dosing protocols (35). Notably, vitamin B6 administration demonstrates significant potential in ameliorating LEV-induced neurobehavioral sequelae, including irritability and behavioral dysregulation. While extended vitamin D supplementation (≥6 months) in DRE patients correlates with reduced SF, caution is warranted: excessive maternal vitamin D intake during gestation elevates neonatal 25-hydroxyvitamin D3 levels, which may correlate with increased risk of childhood epilepsy (particularly cases initially diagnosed between ages 1–4 years).

The risk of epilepsy in people with T1DM is approximately threefold greater than that in the general population. Meanwhile, the incidence of epilepsy in patients with T2DM is approximately 35.0 per 10,000 person-years, and long-term use of enzyme-inhibiting ASMs is associated with an increased risk of new-onset T2DM. Acute symptomatic seizures can be caused by acute alcoholism, alcohol withdrawal and uremic encephalopathy; moreover, the likelihood of epilepsy in individuals with alcohol dependence is greater than that in the general population. Patients with end-stage renal disease undergoing dialysis exhibit a markedly elevated prevalence of epilepsy. New-onset epilepsy in this population frequently presents with aggressive phenotypes characterized by rapid progression and high mortality. Critically, the mortality risk among epileptic end-stage renal disease patients is 1.11-fold higher than in their non-epileptic counterparts.

Epilepsy and metabolism constitute a multidimensional, interdisciplinary research field involving neurobiology, metabolomics, genetics and other disciplines. An in-depth understanding of the causal relationships between various metabolites and epilepsy and an exploration of related mechanisms may provide new targets for epilepsy screening and prevention.
